# Supplementary material for: Complexome profiling of the Chlamydomonas psb28 mutant reveals TEF5 as an early PSII assembly factor
Source: Plant Cell. 2025 Mar 17;37(6):koaf055. doi: 10.1093/plcell/koaf055 (PMC12164590; doi:10.1093/plcell/koaf055)
Supplement: koaf055_Supplementary_Data [file koaf055_supplementary_data.zip › psb28_interactive.zip!/plots/intensity/qualityPlots/peptides/TRK1_Cre02.g080200.t1.2.html]

### Cre02.g080200.t1.2

Trivial name: TRK1  
SeqCoverage [median]: 8.40  
Mass: 77.66 kDa  
  
 

| Sequence | MissedCleavage | Mass [Da] | wtIntensity | muIntensity | Score |
| --- | --- | --- | --- | --- | --- |
| AAAPSISR | 0 | 771.42 | 0.00 | 383.12 | 64.27 |
| ESVLPSDVTAR | 0 | 1172.60 | 0.00 | 1468.60 | 94.69 |
| FGITVNHVVEAAK | 0 | 1383.75 | 448.75 | 946.05 | 50.21 |
| FLAIDAINK | 0 | 1003.57 | 0.00 | 9536.71 | 75.38 |
| HVGIDTFGASAPAPTLYEK | 0 | 1972.99 | 0.00 | 2281.37 | 58.25 |
|  |  |  | 448.7 | 14615.8 |  |
